# LoRaChainCare: An IoT Architecture Integrating Blockchain and LoRa Network for Personal Health Care Data Monitoring

**DOI:** 10.3390/s22041497

**Published:** 2022-02-15

**Authors:** Bouthaina Dammak, Mariem Turki, Saoussen Cheikhrouhou, Mouna Baklouti, Rawya Mars, Afef Dhahbi

**Affiliations:** 1 Department of Computer Science and Information Technology, Applied College, Princess Nourah Bint Abdulrahman University, Riyadh 84428, Saudi Arabia; 2 ISIMG, University of Gabes, Gabes 6033, Tunisia; 3 CES Lab, University of Sfax, Sfax 3038, Tunisia; mouna.baklouti@enis.tn; 4 ReDCAD, University of Sfax, Sfax 3038, Tunisia; saoussen.cheikhrouhou@redcad.org (S.C.); rawya.mars@redcad.org (R.M.); 5 Department of Administrative Sciences, Applied College, Princess Nourah Bint Abdulrahman University, Riyadh 84428, Saudi Arabia; andhahbi@pnu.edu.sa

**Keywords:** healthcare, Internet-of-Things, blockchain, edge computing, fog computing, security, LoRa, IPFS

## Abstract

Over the past several years, the adoption of HealthCare Monitoring Systems (HCS) in health centers and organizations like hospitals or eldery homes growth significantly. The adoption of such systems is revolutionized by a propelling advancements in IoT and Blockchain technologies. Owing to technological advancement in IoT sensors market, innovations in HCS to monitor patients health status have motivated many countries to strength their efforts to support their citizens with such care delivery systems under the directives of a physician who has access to patient’s data. Nevertheless, secure data sharing is a principal patient’s concern to be comfort to use such systems. Current HCS are not able to provide reassuring security policies. For that, one of our focus in this work, is to provide security countermeasures, likewise cost-efficient solution for HCS by integrating storage model based on Blockchain and Interplanetary File Systems (IPFS). Blockchain technology is an emerging solution in pharmaceutical industry and starts to take place for HCS and allows HealthCare providers to track connected devices and control access to shared data, hence protecting patients’ privacy. Furthermore, the addition of Edge and Fog computing has improved HCS to react in real-time and enhance their reliability. A variety of communication protocols can connect sensor devices to edge/Fog layer and the best choice will depend upon connectivity requirements: range, bandwidth, power, interoperability, security, and reliability. Instead, systems efficiency would decline and hurt if communication protocol is inconsistent. LoRa (Long Range) communications technology is emerging as the leader among Low-Power Wide-Area Networks (LPWANs) entering the IoT domain benefiting from many features such as long-range distances and low power consumption. This work proposes LoRaChainCare, an architecture model for HCS which combines the technologies Blockchain, Fog/Edge computing, and the LoRa communication protocol. A real implementation of LoRaChainCare system is presented and evaluated in terms of cost, run time and power consumption.

## 1. Introduction

Over the past few years, different healthcare monitoring systems (HCS) have been developed and proposed; aiming to collect, process, and analyze data acquired from various sensors and devices. These systems are also responsible for monitoring and observing patient vital signs. The advent of Internet of Things (IoT) technologies has also facilitated such progress. In this context, the Internet of Health Things is an area of uncompelling change and expansion in the new age of smart houses, smart cities, and all digital things [1,2]. The cloud computing paradigm has emerged as a solution to develop HCS systems for patients’ monitoring. Such systems include many types of medical sensors such as ECG, blood pressure, and oxygen monitor linked via a network to the cloud for storage and processing in order to allow medical professionals to observe, diagnose and give treatment to patients. In fact, apart the heath legislation and patient rights and responsibilities, many countries have made efforts to establish HCS systems and make health services accessible for all individuals of society. As an example, we cite the Kingdom’s efforts to provide comprehensive health care in an accessible and simple manner [3].

Cloud-based solution faces many design constraints related to network bottlenecks, in particular bandwidth and energy [4], are shaping the design HCS. These constraints are even more serious over the last years, when the amount of data is huge and QoS demands in healthcare systems are increasing. In this context, according to industrial report [5], the connected medical devices market was valued at USD 28.24 billion in 2020, and it is expected to reach USD 94.32 billion by 2026. Edge and Fog computing are considered the key enabling paradigms to solve the network problems of the cloud centralized computational model to more decentralized models. In these models, the edge layer collects medical data from sensors, and transmits them to the fog layer, which in turn performs the treatments and sends the results and observations to the cloud layer via internet. However, both internet and cloud data centers are exposed to security threats. As medical data have an important role in disease analysis and in diagnostic results, securing patients’ health documents is of paramount interest, targeting more than one privacy measures including access control and data integrity.

Recently, a wide range of IoT health applications and frameworks have been developed in the one side to automate and make health services accessible to individuals, and in the other side to facilitate medical data exchange between different actors in a secure and a rapid way. It is to be noted that personal medical systems have many requirements, such as data sharing, data security and consistency, data reliability, and convenience [6]. Traditional healthcare systems are not able to meet these crucial requirements because they cannot ensure the safe use and sharing of data in a secure and a controlled way. Hence, to face reliability, fault tolerance and privacy challenges, Blockchain technology is recently adopted in the medical and e-health domain [7,8,9,10,11,12]. This technology is a secure and transparent distributed ledger, and it paves the way for a revolution in existing healthcare systems by integrating its unique features [13,14,15]. However adopting such a computationally intensive-based mechanism to a resource-constrained IoT devices and ecosystems is a challenging task. In fact, multiple problems arise such as real-time processing, data security and scalability and architectural based problems. Currently, to the best of our knowledge, there are no studies that propose a real implementation of HCS and integrates Fog and Blockchain technologie with a focus on the architectural and scalability issues. For example, in [14], the proposed framework studies theoretically the transmission of health record (HER) between Fog layer and Blockchain and no focus on the architectural and scalabilty issues is presented, nor a proof of concept and framework evaluation have been made. In this context, we propose LoRaChainCare, a HCS prototype model integrating Fog and Blockchain to allow close to real-time data processing and scalable and secure patient’s health. To mitigate real-time requirement, LoRaChainCare integrates Edge and Fog devices supporting LoRa communication protocol which is a relevant communication technology for healthcare systems by which energy and security issue are reliable in addition to a long bandwidth transmission. In addition, LoRachaincare integrates a secure and scalable storage model based on IPFS and blockchain technology.

Blockchain is a decentralized data management platform that provides immutability. Thus, it can perfectly support file traceability metadata on a distributed file system like IPFS, due to the similarity in their structure.

In this paper, we propose a novel IoT architecture integrating Blockchain and LoRa network to monitor personal health care data in a secure and efficient process. Our proposed system is based on a multi-layer architecture to collect data from IoT sensing devices, share it and store it to be analysed and accessible among different actors. The Edge/Sensor computing layer collects data from different sources and send relevant ones to the upper layer which is the Fog layer. This latter is based on the LoRa network. Then, the cloud layer contains all the resources that will manage the recovered data from the lower layer. A single main private permissioned Ethereum is also implemented to assure traceability and security issues of the proposed HCS system. In this context, IPFS is combined with the Blockchain technology to improve the storage of medical records. Finally, a Data monitoring/Analytic layer allows to control and monitor the patients’ health data through mobile or web applications.

This article first aims to review all related background in the context of fog computing, Blockchain technology and IoT healthcare systems. Then, it discusses related work with a particular focus on IoT-Blockchain platforms proposed in the healthcare domain. Finally, a multi-layer IoT-based Blockchain framework integrating the LoRa network, named LoRaChainCare, is proposed. A real case-study implementation is also presented and evaluated in terms of Blockchain resources, data transmission time and power consumption.

In summary, major contributions of this article can be summarized as follows:

1. to present a background of the most prominent technologies related to Home Care Systems mainly: Fog and Edge computing, Blockchain and smart contracts;
2. to discuss and compare the related work dealing with IoT-Blockchain platforms in the context of e-healthcare;
3. to implement a multi-layer architecture based on IoT and Blockchain aiming to collect and secure patients’ data access by healthcare service providers and stakeholders while satisfying security, reliability and energy constraints.

The remainder of this paper is organized as follows. Section 2 introduces relevant concepts addressed in the paper, in particular the basics of Fog and Edge computing, LoRa communication protocol as well as Blockchain and smart contracts. Section 3 discusses literature review. Section 4 presents our design and solution for a new IoT-Blockchain healthcare system. Then, Section 5 highlights the architecture workflow. Section 6 details the implementation process of such HCS system, while Section 7 analyses the obtained experimental results in terms of cost, runtime and power consumption. Finally, Section 8 concludes the paper with a brief outlook on future works.

## 2. Background

The focus of this section is to depict the Edge and Fog computing paradigms. It also presents LoRa communication protocol. Moreover, it gives definition and functionality of Blockchain technology.

### 2.1. Edge and Fog Computing

Edge computing is the deployment of processing and storage resources near the location where data are produced. It alleviates the central clouds work by distributing some computations to processing devices located too close to IoT devices. Such tasks can be data filtering, analysis, or signal converting. The local data processing vastly reduces the transmission rate, occurring far less bandwidth and service delay than might otherwise. The Edge computing integration becomes more important in healthcare systems [16,17,18] by empowering it to react as fast as possible in emergency situations.

Fog is first introduced by CISCO, and it refers to the distributed infrastructure between the Edge device and the cloud. It includes Fog nodes and the necessary network connections for transporting the data. Any device with computing, storage and network connectivity come under the definition of fog nodes [19,20,21]. Fog nodes allow data to be collected and processed locally and reduce recurring transmissions over the cloud between Cloud and Edge devices. The proximity of Fog nodes to both IoT devices and end users ensures real-time computation and improved QoS [19,22].

### 2.2. LoRa Communication Protocol

LoRaWAN technology offers a long-range transmission of low data rate with optimized power consumption and a reduced cost. Indeed, data is transferred from the end device (sensor node) to the LoRa gateway using free unlicensed frequency within the ISM band so that it can be used, safely, for the e-health applications. Frequencies are ranged from 868 MHz to 900 MHz. LoRaWAN is able to transmit data up to three miles (five kilometers) in urban areas, and up to 10 miles (15 km) or more in rural areas. The other main feature of the LoRaWAN is the low power consumption measured in milliwatts (mW) so that some battery-operated devices can last for up to 10 years. LoRaWAN is deployed in a star topology which is adapted for applications that require long-range communication between a large number of devices that exchange small amounts of data (Data rate depends mainly on the spreading factor). All the LoRaWAN features are detailed in [23].

The components of a typical LoRa-based architecture and are as follow:

- The end devices: are the LoRa nodes which send the data to the Gateways and they are classified into 3 classes (A, B or C) depending on the node availability and its power consumption.
- The LoRa server: handles the entire network including the up-link and down-link communications coming respectively from the end device to the application server and from the application server for the end devices. In the other hand, in this server, several encryption/decryption processes are performed to ensure the data security.
- Gateway: is the bridge between the end devices and the LoRa server. Three frequency channels and Internet protocol are supported to communicate respectively with end devices and LoRa server.
- Join server: checks for the end device authentication to send encrypted data through the LoRa network. The authorization is made through an activation process; Over-The-Air-Activation (OTAA) or the Activation by Personalization (ABP). The encryption is performed by the join server generating two session keys (NwKSkey and AppSkey). Using the ABP technology, the session keys are set statically in the end device and the network server, and they are not modified at any time. In the OTAA technology, the generation of the session keys is done dynamically at each device re-connect or reset. Consequently, the OTAA technique guaranties a better security in the data transmission between the end device and the network server.

### 2.3. Blockchain and Smart Contracts

Blockchain, referred to as Distributed Ledger Technology (DLT) for transactions [24], enabling all participating nodes to validate transactions related data, ensuring thus unalterable and transparent history of any digital asset. It eliminates the need for third-party verification solving thus the problem of trust for sensitive data. All nodes of the network agree at the ledger to put each transaction into a block and validate that block with public key cryptography to append it to a chain. This validation process is referred as mining. All miners maintain a copy of the entire chain of blocks. The resulting list of blocks is chained using hash pointers and creates thus a tamper-evident data structure or log that helps to track data provenance and traceability abilities. Indeed, the potential of Blockchain is due to Smart contracts, which are algorithmic program codes, that can execute aunonomously to perform transactions in Blockchain. They replace third parties that centralize and manage the distributed ledger. All smart contract executions are recorded on the Blockchain, and made therefore publicly visible by all nodes. Additionally, Blockchain can help to decrease time and costs of information systems. The security of data in Blockchain is ensured by its distributed data storage, secure protocols, and mainly its consensus mechanism.

## 3. Literature Review

Different state-of-the-art eHealth architectures [25,26,27,28,29,30,31,32] have incorporated Fog and Edge computing to make the processing and access of health medical data faster.

In [30,31], authors considered hierarchical infrastructures composed of four layers: IoT layer, Edge layer, Fog layer, and Cloud layer. In [31], the mobile device receives health data from user’s sensors and provides the environmental and geolocation information to send them along to the Fog node. In [30], the authors proposed Mobi-IoST, to address the problem of a seamless connectivity between IoT devices and cloud servers due to devices mobility.

In [29], the authors propose fall detection system architecture integrating smart LoRa-based Edge gateways (Edge layer) and LoRa access points (fog layer). In their work, health and contextual data are sent using Bluetooth Low Energy (BLE) to a LoRa gateway for compression and forwarded later to the LoRa-based access point to RNN processing (recurrent neural network) and distributed storing. Although system implementation is presented, the authors only evaluated the precision of implemented RNN with no evaluation considering Edge and Fog performance.

Although the aforementioned works have explored Fog and Edge computing for rapid access and processing of data in healthcare systems, a weak interest is accorded to security and privacy of patient’s data. Indeed, the distributed topology of Edge/Fog computing increases the need for securing the network against the attacks.

Blockchain is a cutting-edge technology, which is gaining considerable interest, and integrated in many works [13,15,33,34,35] to cope with securing stored data and communication within network. In [34], the authors propose a three-layer architecture where a Fog layer is integrated between the healthcare sensing layer and cloud data centres. They integrate a Blockchain model at the edge of the network that offers trust access and control over the network. Each Fog node is a collection of blocks, smart contracts and ledgers. In addition to endorse security, Blockchain technology allows for storing the records of transactions between the different entities. In [35], the authors proposed FogBus, a platform independent framework, and presented a cost-efficient prototype for Sleep Apnea patients. The framework integrates both blochchain and Fog computing. Fog Gateway Nodes are connected to sensing layer via wireless/wired communication protocols such as Zigbee, Bluetooth and NFC. Fog Computational nodes (FCNs) are provided with processing cores, memory and bandwidth so that they are able to execute the back-end program of the applications. Some FCNs are equipped with adequate security features and fault tolerant techniques such as Blockchain and replication. In [13], patients’vital signs are remotely being monitored by an immutable history log, providing thus a global access to medical information. The focus of this work is the conceptual design of health data sharing systems based on Hyperledger Fabric Blockchain-based smart contracts. Four layers compose the developed system: connectivity layer (routing management, security management, message brokers and network management), IoT physical layer of health (health devices), Blockchain IoT service layer (Blockchain-related service), and application layer (user interaction). Sensor data are sent by the gateway to the IoT server, which in turn, routes them to the Blockchain where they will be saved in the WorldState (off chain). In [15], Ray et al. investigated the integration of IoT and Blockchain for e-helathcare and discussed their features to harness the IoT-centric health services. They proposed an IoBHealth data-flow architecture for integrating Blockchain and IoT sensory data collected from patients to be securely accessed and managed by healthcare service providers and stakeholders. The proposed IoBHealth system is developed as an improved version of the existing MedRec architecture [36]. Its ultimate aim is to transmit and manage the electronic health record (HER) in a decentralized way and to guarantee its security, immutability and transparency between the different stakeholders of healthcare industry. However, no experimental evaluation, nor a comparison to prior work have been made. In [14], authors proposed a Blockchain leveraged decentralized eHealth architecture integrating Edge and Fog devices and using Bluetooth or ZigBee protocol for data transmission. The Fog devices run a Blockchain protocol. To cope with the problem of large data storage on Blockchain, some works [37,38,39] merge smart contracts with decentralized peer-to-peer Interplanetary File System (IPFS), which relies on a global Distributed Hash Table (DHT) to provide HCS records sharing in IoT scenarios. These works leverage IPFS technology to improve data sharing and IoT communication in untrusted environments.

The aforementioned works gave us a better understanding of main challenges in existing HCS architectures. In Table 1, we give a summary of techniques or methods adopted in the reviewed papers. We note that the concern in these papers is to bring performance and security to healthcare systems. However, considering all reviewed paper in Table 1, they fall into at least one of the following limitation: (i) papers do not justify the means by which they choose the communication protocols to use between IoT layer and Fog/Edge layer; and (ii) papers do not integrate Blockchain solution in their methodology and/or in their evaluation; and (iii) articles do not consider the problem of large data storing over Blockchain or cloud; and (iv) papers do not provide a system implementation.

## 4. Architecture Description

In this section, we describe the proposed architecture and we introduce the concept of data transmission between layers. The proposed system contains superposed layers in order to route the collected data from the lowest layer to the highest one. Using a multi-layer system offers the following advantages:

- The modular aspect of the system facilitates the integration of several functionalities. The upgrade and the maintenance of sub-systems doesn’t affect the entire architecture
- Designing highest layers is carried out with abstraction of the lowest ones. This allows to discard the hassle of designing hardware components when developing the software applications.
- Ensure the interoperability between heterogeneous components. In fact, the system contains several components with different technical characteristics and connectivity parameters. This heterogeneity is absorbed and supported in the lowest layers so that the proposed applications will be addressed to all of them.

In the following section we describe the proposed architecture layers as well as the participating actors.

### 4.1. System Actors

**Figure 1:**
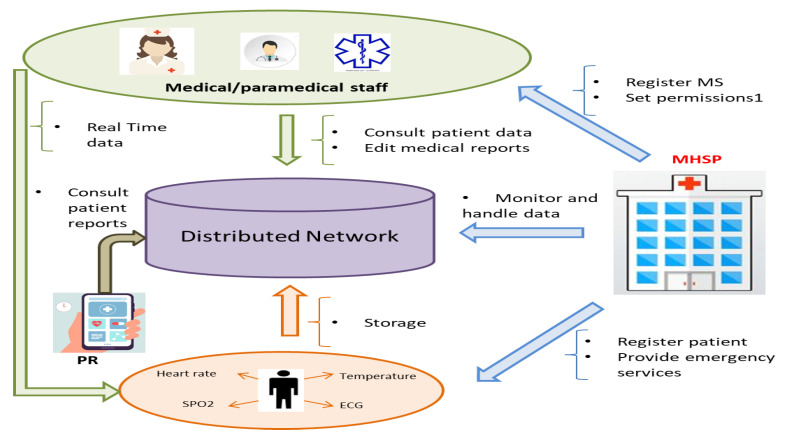
System Actors.

In the proposed architecture, several actors intervene to handle the patient data recovered from the sensor layer. Main actors are presented in Figure 1.

#### 4.1.1. Main Healthcare Service Provider (MHSP)

This actor can be mainly a hospital administrators which monitor and handle all the patients data, but also the other actors permissions. Indeed, the MHSP registers all the patients with their relative data (Etehreum address, Name, Age, Phone number, the doctors allowed to consult the patient data). When a patient needs an emergency service, the MHSP is responsible to provide and to put in place the needed materials. Finally, the MHSP registers the system actors with relative permissions.

#### 4.1.2. Medical Staff (MS)

The medical staff represents any medicine practitioner working in the healthcare system mainly including doctors, nurses and some paramedical persons. These well defined MS can interact with their Patient’s medical records. In fact, each MS has specified permissions related to the patients data. For example, doctors are allowed to monitor stored data of their patients, as well as real-time data in order to edit a medical report about the patient’s condition. Nurses are allowed to monitor the stored or the real time data. And Finally, the paramedical staff is allowed to consult some required actions related to patient (transferring patient with an ambulance, cleaning patient room, etc.).

#### 4.1.3. Patient

Patient actor represents any person receiving or registered to receive medical treatment. To guarantee the user’s self-sovereignty, while ensuring the patient’s right to privacy, the patient could select the medical staff that could access to his personal data. Therefore, he is allowed to monitor all his data and especially the doctor’s reports to follow up the health and the treatment evolution.

### 4.2. Architecture Infrastructure

In order to ensure the best system requirements, it is necessary to implement an appropriate infrastructure.

The infrastructure of the proposed approach contains a set of superposed layers which are described below.

#### 4.2.1. Edge/Sensor Computing Layer

The Edge/Sensor computing layer is the point of contact with patients, collecting their health data and exchanging messages and communicating with devices for further monitoring. It provides also protocol interoperability by supporting various protocols and standards. The data collection is ensured through several sensors that are passive components used to collect all the data about the patient’s health. Many parameters could be measured such as temperature, $SPO_{2}$, heart rate… In addition, this layer contains some actuators which receive the commands from the corresponding actors after the data analysis. For example, when the data collected from the sensors indicate that the patient need some treatment, doctors can give commands to the syringe driver to push the appropriate quantity of drug.

These Sensors are connected to processing nodes. Each node analyzes, locally, the recovered data so that it can select the data to send to the next layer. Indeed, the patient’s parameters are sensed periodically and sometimes there is no change between two successive measurements. Consequently, it would be useless to send the same data to the Cloud/Blockchain and to support extra fees to save insignificant data. This solution allows optimizing the power consumption (since the sensor node consumes more energy when using the wireless communication). Moreover, the proposed solution optimizes the use of the cloud and Blockchain resources.

#### 4.2.2. Fog/Network Layer

This layer includes all the gateways which receive the data from the Edge layer using the LoRa network. Then the gateways transmit this data to the cloud layer using the wifi network in order to be analyzed. In fact, LoRa is a Low Power Wide Area Network (LPWAN) which offers an optimised power consumption but with a limited data throughput (1%). The end device sends data, using connected LoRa Antenna, to the LoRa Gateway through an available channel. A LoRaWAN Gateway supports at least 3 frequency channels with a given bandwidth. For example 868.10 MHz, 868.30 MHz and 868.50 MHz are supported by all the gateways with a bandwidth of 125 KHz.

The same end device is able to communicate with several gateways not only with an exclusive one. After each transmission, this node changes the used channel randomly.

After receiving the data, the gateway sends it to the LoRa server using the Internet Protocol. If the LoRa server receives the same data of the same end device through several gateways, it eliminates duplication thanks to a frame counter included in the exchanged payload. This frame counter is used also to reduce the risk of message being spied and re-injected in the LoRa network by a hacker.

#### 4.2.3. Cloud/Server Layer

This layer contains all the resources to manage the recovered data from the Fog layer. It includes mainly the LoRa server, the Join server and the application server. In This part, we will merge the LoRa server and the Join server as they manage all the LoRa network including the exchanged data and the activation process. In this work we used the OTAA process to allow the end device to integrate the LoRa network. Indeed, the security issue of the OTAA activation process is better than the ABP’s one.

#### 4.2.4. Blockchain Network

In our proposed model, we implement a single main private permissioned Ethereum Blockchain to monitor the healthcare data of patient in HCS while ensuring his security and privacy. In this network, participants may be known in advance and may be partially trusted where smart contracts are used to manage interactions and roles agreed between actors, and thus ensure the patient data privacy.

#### 4.2.5. Data Monitoring/Analytics Layer

This layer contains sevaral tools to control the patient data:

- An off-chain InterPlanetary File System (IPFS) is used to store the user’s personal data, such as the license of participation in the drug supply chain, ensuring the reliability, accessibility and integrity of the stored data. Data integrity is preserved by generating a unique hash (i.e., the SHA-256 hash of the data) for each uploaded file on its server, where only the IPFS hash, which acts as a pointer, is stored on the Blockchain and accessible by the smart contract, and any changes that occur in any of the uploaded file are reflected in the associated hash. In particular, the encrypted participation license can be stored separately from the Blockchain in an off-chain storage (e.g., through a private network of nodes, which is either joint or disjoint from the Blockchain network), while the Blockchain only stores a pointer to the encrypted data residing on this off-chain storage. This allows our proposed system to ensure the security and privacy requirements, since IPFS hashes act as control pointers to encrypted data.
- Web Application: This application is used to manage all the actors permissions regarding the patient data. Each of these actors is able to authenticate through this web application in order to communicate with the Blockchain network and the patient data.

## 5. Architecture Workflow

**Figure 2:**
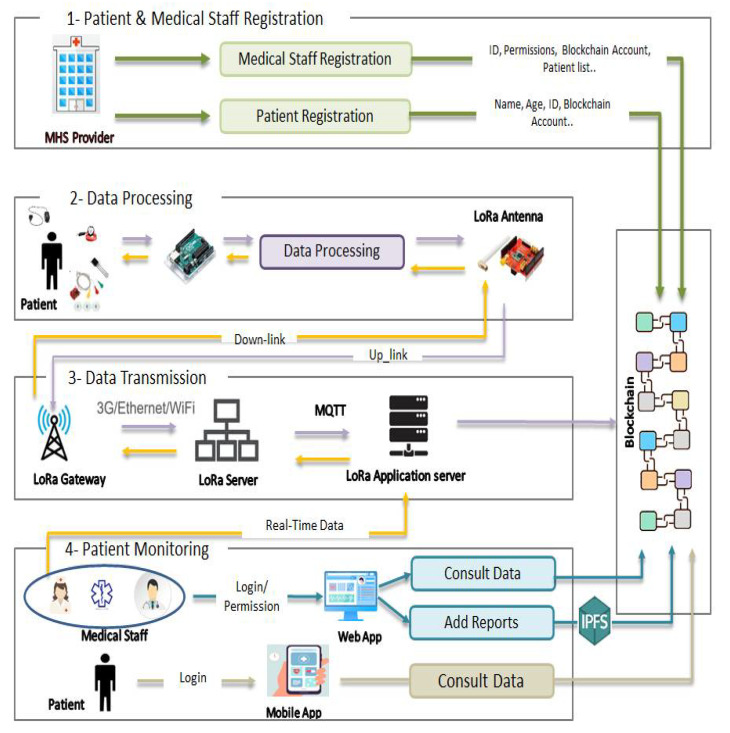
Proposed Architecture Workflow.

In this section, we describe the architecture workflow and the different functionalities of the proposed system. Like illustrated in Figure 2, the architecture workflow could be modeled with 4 parts. All these parts communicate together using exchanged data.

### 5.1. Patients and Medical Staff Registration

Before starting the data monitoring and storing processes, all the active actors should be registered in the main system. The MHSP (Main Healthcare Service Provider) is responsible to register all the patients who benefit from the hospital services. The MS (Medical Staff) is also registered to ensure the different hospital services.

- Patients Registration: It includes the registration of the hospitalized patients, but also the elderly persons staying home and who’s health state is monitored by the hospital’s medical staff. All the patients parameters are stored in the Blockchain such as the Ethereum address which used as an ID, Name, Age, phone number, etc. Each patient is affected to a corresponding MS to manage all the patient requirements. For example, one or many doctors should monitor his health state, give the corresponding diagnosis and set reports describing the proper treatment. Patients are able to authenticate in the proposed system and they are authorized to consult their own data.
- Medical Staff Registration: The MS includes doctors, nurses and paramedical staff. The MHSP register each one of them and set their personal parameters like Ethereum address as an ID, Name, Role, Licence ID, etc. The most important thing is to affect the right permissions to each one of the MS since the proposed system is an access based control. Doctors are able to consult the historical data of the patients to which they are assigned. They are also authorised to ask for real time data before writing a report describing the health state and prescribing the right treatment.
  Nurses are allowed to consult the historical and real time data of patient to which they should carry on. They are able to request for a doctor intervention in case of critical condition thought a web interface.
  Finally, the paramedical staff are allowed to view all the MHSP requests related to a given patient, such as preparing a specific meal to a patient, transferring a patient to the radiology department, taking samples from a patient to the analysis laboratory, etc. Each patient is selected with his ID so that all the needed parameters are shown to the requested Medical Staff.

### 5.2. Data Processing

After registering the patients, specific sensors related to their disease are used to measure all the needed parameters such as temperature, $SPO_{2}$, ECG, blood pressure, sugar, etc. These sensors are connected to a processing platform which performs three actions:

- Data Acquisition: The processing platform recovers data from several heterogeneous sensors (digital, analog, serial...) so that it converts the data to a comprehensive information.
- Data Processing: Classical systems proposed in the state of the art save all the recovered data in the off-chain data storage or in the cloud or Blockchain platforms. For the off-chain servers, data could be modified or deleted due to malicious actions (for example in the case of death that is due to medical malpractice). For the Cloud/Blockchain platforms, data storage requires resources fees. Thus, if the data is stored periodically (Every 1 or 2 min...), it may cost huge expenses especially when some data is insignificant (For example: The same “normal” temperature all the day). Therefore, in the proposed architecture, several processing tasks are performed. Each parameter is analysed and compared to given thresholds. Only values that exceed the thresholds will be stored in the Blockchain platform. Algorithm 1 shows a heart rate processing pseudo code. In this algorithm we studied the variation related to the patient’s age. To do so, we used the traditional formula proposed by Fox et al in 1971 [40]. However the maximum heart rate can depend on other parameters like the patient’s sex, diseases, activities, the drugs he takes.
- Data Modulation: After analyzing data and decide which one will be stored in the Blockchain platform, the processing platform integrates a LoRa shield to ensure the modulation then the transmission of the data with the LoRa protocol.

**Algorithm 1** Heart rate Analysis.
 heart_R← Read heart rate from sensor
 age← Read age from Blockchain               ▹ Using the patient ID
 **Function** heart_rate (heart_R: Integer, Age: Integer): **Boolean**
 Store←False
 **if**
(heart_R≥120−age) or (heart_R≤60) **then**
     Store←True
 **end if**
 heart_rate←Store
 **EndFunction**

### 5.3. Data Transmission

The LoRa Shield sends the modulated data through the integrated antenna to the gateways whose ranges cover the considered end device. Indeed, the LoRa device is not registered in a specific gateway but into a given LoRa server, so that the patient data can reach several gateways in order to find an available channel. This data is demodulated in the received gateway then sent to the LoRa server using the internet protocol. The transmitted frame includes mainly the encrypted data using the AppSKey, the MIC field for the end device authentication and the frame counter which is used for security purposes and data duplicate elimination.

The data sent from the end device to the LoRa server is called up-link message. The data sent from the Application/LoRa server to the end device is called down-link message.

Finally, the data reaches the application server. Many communication protocols could be used such as the MQTT (Message Queuing Telemetry Transport)and the HTTP POST. In the proposed architecture we used the MQTT protocol. Indeed, MQTT is a light protocol based on the publisher-subscriber relationship instead of the client-server one. Consequently, the application server has no longer to request data when it has no idea when it will arrive. Especially in our proposed systems, the data are not sent periodically, but it depends on the data processing performed in the end device. Patient data will be transmitted to the subscriber client (Application server) as soon as it arrives to the Broker.

This application server could be used to:

- Store the patient data into the Blockchain platform.
- Recover patient parameters from the Blockchain (for example the Patient Age).
- Send user commands (patient or Medical Staff) to LoRa Devices requesting for real time data (Down-link Stream).

### 5.4. Patient Monitoring

One of the main purpose of the proposed system is to ensure the patient data monitoring. In Fact, this data can be monitored through mobile application or Web application.

- Mobile application: is used by the patient himself to consult his own data. After the patient authentication, he is able to discover all about his health state (list of assigned doctors, the diagnosis, drugs, doctor’s reports...) according to the permission given by the MHSP.
- Web application: is used by the Medical Staff to monitor the patient information. Indeed, after the authentication, each one of the Medical Staff is able to handle all the application functionalities according to the permission given by the MHSP. For example, doctor can consult all the patient data which are assigned to him and whose parameters are stored in the Blockchain platform. He can ask for realtime parameters using the LoRa down-link communication through the LoRa application server. Finally, he is able to write a report about the patient health state. This file will be uploaded by the doctor and stored into the IPFS platform. The Hash of the uploaded file is then added to the Blockchain platform.

Sensors are connected to the Arduino Uno platform which processes the data including data acquisition, data conversion, checking whether measurements are in normal or abnormal range and data modulation. Data modulation is performed to send patient data to the LoRa Gateway using the Arduino connected to LoRa Shield.

## 6. Implementation Process

Our implementation environment is based on a case study in which a patient, registered by the MHSP, is equipped with vital-signs sensors to monitor his health data by the MS.

In LoRaChainCare, data communication is ensured from the lowest layer (IoT end devices) to the highest one (application and Blockchain platforms) thought the Fog-based LoRa gateway. The IoT devices consist of health sensors such as ECG sensors, body temperature sensors, SPo2 and heart rate sensors, etc., as well as environmental sensors used to control the status of hospitalization rooms. The LoRa gateway is responsible for processing requests (Joining) and providing the required sensors reading to the MS. The Gateway sends the uplink data to the LoRa server and the Application server using Internet Protocol and MQTT protocol respectively. Finally, data is stored in the Blockchain platform. The proposed system uses the Ethereum Blockchain network [41] which uses smart contract in order to make transactions and store data on the Blockchain’s registry. This is achieved through the Solidity computational programming language, that stores smart contract programs in the form of Ethereum Virtual Machine (EVM) bytecode, and enables the execution of transactions in the form of function calls within that code/program. The smart contract is a collection of code (its functions) and data (its state) that resides at a specific address on the Ethereum Blockchain.

User accounts can then interact with a smart contract by submitting transactions that execute a function defined on the smart contract. Likewise, transactions are logical operations defined in the smart contract that can interact with assets. The transactions are responsible for modifying the value of participants and assets in the Blockchain network.

Some account permissions allow user to submit files and reports into the IPFS distributed platform then store the Hash into the Blockchain in order to reduce the gas fees.

The implementation of the proposed system is divided into several interactive sub-systems including mainly the hardware part which, includes the end nodes and the communication protocols and the software part which, includes Blockchain, smart contracts, web and mobile application.

### 6.1. Hardware Implementation

**Figure 3:**
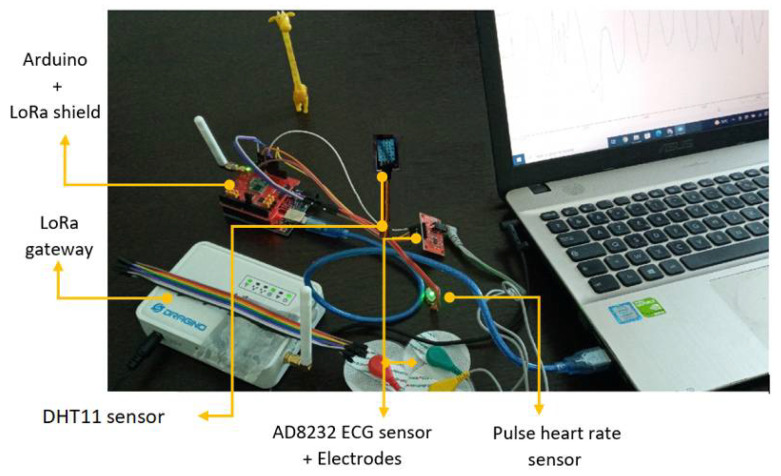
Developed system for LoRaChainCare.

The hardware implementation consists of connecting sensors to Arduino Uno as well as establishing communication with LoRa gateways. In Figure 3, we present the developed LoRaChainCare system. For the LoRa network we used the Dragino Single Channel V2 kit which includes the LG01-N gateway and the LoRa shield based on the sx1276 transceiver.

For the health monitoring, we used 2 healthcare sensors: ECG(AD8232) and Pulse heart rate sensor (SEN-11574). For the patient room control, we used one environmental sensor—DHT11 which measures the room temperature and humidity. The voltage supply of these sensors is equal to 3.3 V. All these sensors are connected to an Arduino Uno platform which is connected also to the LoRa shield.

Table 2 gives a summary about the used sensors. The normal ranges are set by the MS depending on the patient parameters like (age, deceases, gender...). In the case of the ECG monitoring (AD8232), the sensor reports the heart activity which will be processed by the Arduino platform to compute the Heart Rate variability (HRV). The heart rate sensor measures the number of heart beat per minute and finally, the DHT11 sensor measures the room temperature and humidity. If the readings exceeds the threshold values, detected values will be transmitted through the LoRa network to the Blockchain platform and the MS will be notified. Abnormal values indicate an urgent health status such as bradycardia and tachycardia.

The different vital-sign readings can be visualized using serial plotter which is an Arduino IDE tool.

**Figure 4:**
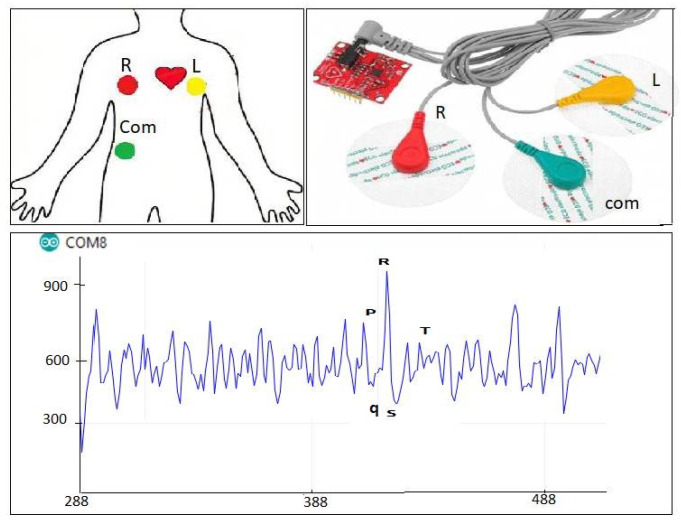
Visual Patient’s ECG sensor readings.

In Figure 4, we depict the acquired ECG signal of the electrodes through the AD8232 sensor. As shown, this signal is a series of a *P* wave, QRS complex, and a *T* wave. The *P* wave indicates atrial depolarization, the *PR* interval represents the time during which a depolarization wave travels from the atria to the ventricles and finally *R* peak counts the heart beats per minute. The period between two successive *R* peak determines the *PR* interval.

The system will contribute towards possible cardiac abnormalities detection in case of higher beats per minute ( high *R* peak ). Similarly, a *PR* interval variations indicates a cardiac anomalies.

### 6.2. Software Implementation

In this section, we present the HCS Blockchain implementation using Ethereum and the development of the web-based user interface designed to interact with the HCS Blockchain services.

#### 6.2.1. Smart Contract Implementation

The smart contract algorithm, includes actors and transaction descriptions to be established in the proposed healthcare Blockchain. Also, we demonstrate how the exploited smart contracts were built with specific reference to the data structures and their interfaces.

In our system, we designed and implemented all the different functions within one single smart contract written using solidity language and deployed on Ethereum Blockchain. Table 3 summarizes the different actors, assets and transactions for the smart contract of the proposed system. The actors includes MHSP, doctors, patients, and nurses, whereas the assets are health sensors and related readings. Thus, a structure data type is used to represent each actor in this proposed system. An effective data extraction through a mapping of a key-value pair is provided by Solidity. Other members of the structure, such as address, string and unsigned integer are used to represent different actor’s information. A mapping variable for each actor is defined with actor’s address (in the form of address datatype) as keys pointing to the corresponding actor’s structure. A code snippet for the described data structure of patient is provided in Listing 1. Also, to increase security of smart contract against unauthorized access, we implement an access control permissions to control the functions that restrict the access to specific actor. Solidity provides a simple way using modifiers. A modifier allows controlling the behavior of smart contract functions. Listing 2 presents a code snippet for the modifiers that are used to restrict access to patient only.

**Listing 1.** Related data structures of patient.

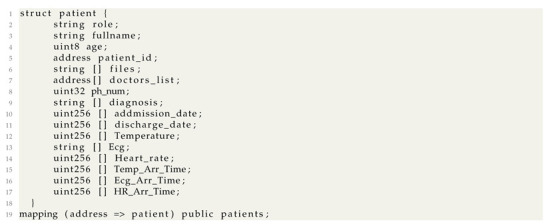



**Listing 2.** Modifier of patient access control.

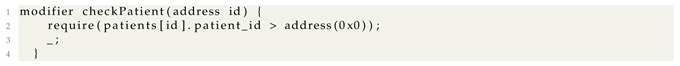



Listing 3 presents two functions of the scenario of secure patient’s health monitoring from sensors based on Blockchain. The MHSP registers the Patient and Doctor in the Blockchain network and passed as an input the required information, such as the Ethereum address, full name, etc. Thereafter, the received data is well saved in the Blockchain ledger. Then, the patient grants the access to get his vital-sign information using healthcare sensors and to his medical information to a tier of the doctor. Therefore, this doctor is allowed to monitor the vital-sign information of this patient based on the functions getECG, getTemperature, and getHeartRate. The full Smart contract code is publicly available in a Github repository (https://github.com/RawyaMars/LoraChainCare/blob/main/contracts/healthCare.sol, 22 Decembere 2021).

**Listing 3.** Patient and Doctor registration Smart Contract functions.

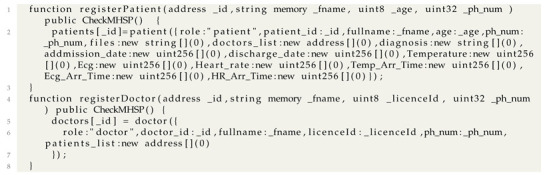

A Smart Contract Development Environment

In this work, we employed an Ethereum-based private network, where the Solidity programming language, developed by Ethereum, was used for implementing the Smart Contracts. At first, we designed and implemented all the different functions within one single smart contract. At this phase, we used a Solidity compiler, called Remix IDE [42], which allows testing simple transactions for correctness and eliminating bugs. In the second phase of code development, we use a development environment and testing framework for Ethereum, named Truffle, which is used also for the compilation and migration of the Smart Contracts. Truffle is a stronger development environment compared to Remix as it not only serves as a Solidity compiler but also allows flexible ways for testing smart contracts by supporting different test environments.

#### 6.2.2. Web Application Development

**Figure 5:**
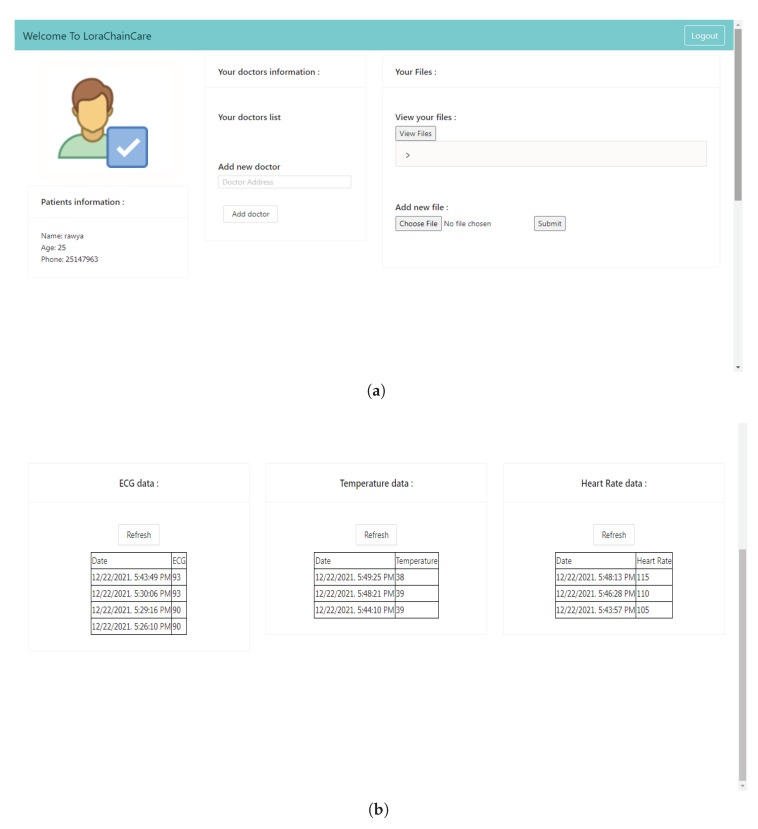
Web-based patient interface of the HCS Blockchain implementation. (**a**) Patient information and functionalities; (**b**) Patient monitored health care data.

Once we completed a major development task on Ethereum’s smart contracts and their implementation in the truffle project, we reached another major challenge. In order to implement the described architecture, we developed a web application that would be based on ReactJS [43] and represents all involved actors sides. React is a free and open source front end JavaScript library enabling to build modern web applications. It was originally developed and maintained by Facebook and its community. It is a versatile framework, and it also has a short learning curve and allows for easy, testable and maintainable code. Since it is the most interactive user interface, we have chosen to work with it. The user can easily interact with the reactJs application interface where both the creation and deployment of the contract is done. To retrieve information about the status of the Blockchain and smart contracts, this ReactJs web application uses web3.js API, which is a JavaScript library provided by Ethereum. The web3.js library offers developpers the ability to interact with Smart Contracts through a HTTP or IPC connection. Hence, we also used the Metamask [44] browser plugin to authenticate and interact with Smart Contracts in the browser. Figure 5 shows the web-based user prototype of the HCS Blockchain implemented using Ethereum. The figure interface shows the different information and functionalities of the patient, as well as patient healthcare data. The Figure 5a shows the patient information and the patient’s functionalities including granting access to doctor and adding medical reports. Figure 5b shows the patient’s records of the health care data.

## 7. Evaluation

To evaluate the performance of the proposed system, we used Ganache tool [45] to test the functionalities of the implemented smart contracts. Ganache represents a personal Blockchain for distributed application development on Ethereum. Ganache automatically generates a set of private and public keys for each account with balance of 100 ETH (Ether—a currency for transaction on Ethereum platform) and used for experimentation along with the simulated node role. We used Ganache to conduct the performance of the designed system in terms of cost analysis, runtime analysis, and power consumption analysis. The Metamask browser extension was configured and connected to all these nodes on the local host to the port 8545. The truffle was also configured over the same port in order to talk to the Blockchain.

### 7.1. Cost Analysis

We provide a Cost Analysis of Ethereum smart contract code and function calls. To perform a transaction on the Ethereum Blockchain, there are fees required to be send to the Ethereum Blockchain, and those fees are what is called gas. A very useful and easy-to-use way for estimating execution and transaction costs, which are key types of costs, is offered by the MetaMask wallet. The execution cost represents costs associated with performing various functions of the smart contract, whereas the transaction cost considers several factors such as the deployment of the contract and the data sent to the Blockchain network. As an illustration, Table 4 highlights the gas cost of each function implemented in the smart contract, along with the costs converted to fiat currency (USD). In Solidity, not all functions require a charge in gas for execution, indeed, only write transactions in the Blockchain require gas. This means that calling a contract to view data (e.g., a balance, contract variables, requesting data) does not require paying a gas fee. Table 4 demonstrates a very low cost of the functions in USD. The highest cost function is the *addFileByDcAndMHSP* function that is executed by the doctor and MHSP. Its relatively high cost could be explained by the change of different variables in the function that requires storage, which are the IPFS hash file and the patient address. Conversely, TemperatureSensor and HeartRateSensor functions show the lowest cost, since they only send the value of temperature and the heart rate pulse to the Ethereum Blockchain. From the observations shown above, it is evident that gas charges are proportional to the number of times the state of the smart contract has changed, also indicating that the storage can significantly raise costs, therefore it is critically important that the user uploads the correct details, as there is no way to reverse the function once it has been executed and the gas charges are permanently lost.

### 7.2. Runtime Analysis

**Figure 6:**
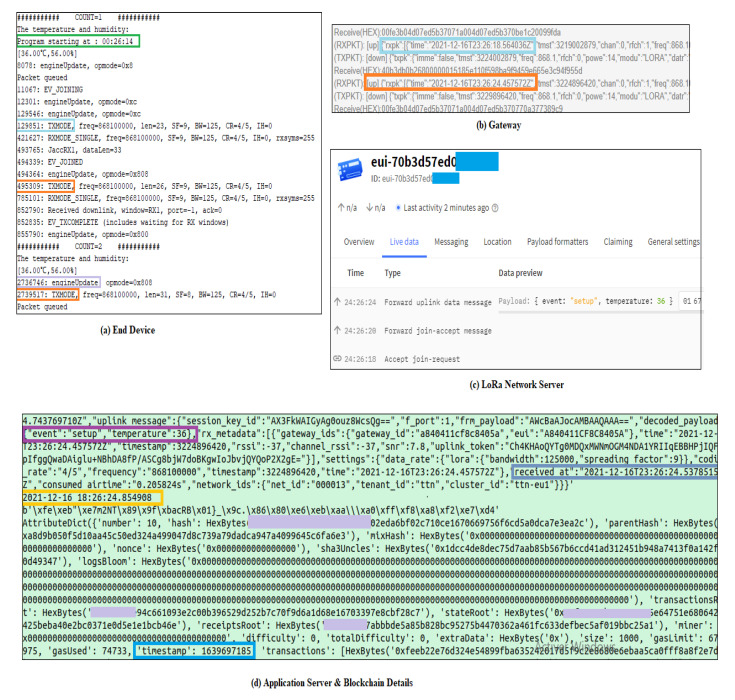
Transmission Time Determination.

The proposed system was implemented and evaluated according to the runtime which means the transmission time from the end device to the application server and the Blockchain platform. For this purpose, we performed several experimentations to accurately evaluate the runtime in each layer. Figure 6 shows the performed processes to compute the transmission time through each level.

Figure 6a shows some time details about the processing tasks in the end device. It includes the program starting time, the time of the end node join request, join accept receipt, the payload transmission, etc. These Times are represented in os_systicks which means the number of systicks since the program starting time. In the LMIC (LoRaMAC-in-C) configuration, the OSTICKS_PER_SEC is equal to 64,516 so that one second corresponds to 64,516 systicks. When the program starts. The end node has to send a join request to the LoRa server to be able to send the patient data through the LoRa network, the Join request process starts after 11,067 systicks next to the beginning of the program and the request is sent effectively to the LoRa server at 00:26:16.01 (starting time + 129,851/64,516). All the times in Table 5 are computed in the same way.

Figure 6a presents two separate processes: the Join request and the patient data transmission. The Join request is performed only one time after the end device reset. Once the end device is recognized by the LoRa network and the keys are generated, the patient data will be continuously sent through the LoRa network with a duty cycle = 1%. The Join request is processed in a total of 494,339 − 11,067 = 483,272 = 7.49 s.

Now the total data transmission time between the end device and the Blockchain platform is determined using Equation (1)

(1) $$T_{tot}=T_{ED}+T_{LoRa}+T_{S}+T_{B}$$

with $T_{ED}$ corresponds to the time spent by the Arduino platform to prepare the Frame (data encryption, headers...). Here, we are not considering the data acquisition from the sensors and the data analysis. $T_{LoRa}$ is the time spent in the wireless communication through the LoRa Gateway. $T_{S}$ corresponds to the runtime into the LoRa Server and the Application server. Finally, $T_{B}$ is the Blockchain data storage time and it is expressed in timestamp. According in Figure 6a–d,

$$T_{tot}=0.03+2.86+0.32+0.15=3.36\;s$$

These times are reported for a temperature transmission (integer) through the proposed system. The Frame counts for 26 bytes including the encrypted payload and several other parameters to be transmitted through three protocol layers (physical, MAC and application).

The wireless communication corresponds to 85.11% of the total transmission time. It includes The LoRa frequency based communication between the end device and the Gateway as well as the Internet protocol communication between the Gateway and the LoRa server.

The $T_{B}$ is relatively small (4.4% of the total time) since we made experimentation in a local Blockchain platform using Ganache environment. This Time could be considerably higher if we target a public Blockchain platform.

### 7.3. Power Consumption Analysis

The main advantage of the LoRa Network is the reduced power consumption compared to other long range networks such as 3G/4G. LoRa is called an LPWAN which stands for Low Power Wide Area Network. When registering the end device into the LoRa server, it is possible to choose one of the three LoRa classes of an end device which are A, B or C described previously in Section 2.2. In this analysis, we adopted the class A node and we measured the power consumption of the several performed tasks. Table 6 shows the characteristics of the sx1276 LoRa transceiver which is used in this experimentation [46].

Table 7 shows the power consumption of the Atmega328p micro-controller as well as the used sensors [47,48,49,50].

In Order to estimate the power consumption of the end node, we consider the following facts:

- The Atmega328p processor is active during all the performed tasks
- The Atmega328p processor goes in the Idle mode after the LoRa Rx window.
- In this section, we consider the Class A node where the Acknowledgment is received in the first Rx Window.
- We suppose that the measurements are done each 60 s and all the data is sent to the LoRa server.

Taking account of these hypothesis, the power consumption at a given time is represented by Equation (2).

(2) $$P_{tot}=P_{proc}+P_{meas}+P_{Tx}+P_{Rx}$$

where $P_{proc},P_{meas},P_{Tx},P_{Rx}$ are respectively the power consumption of the processor, the measurement functions, the transmission and receipt. According to the time measurements performed in Figure 6 and Table 5 and the power model studied in [51], we reported the power consumption and the dissipated power for each task in Table 8.

Table 8 shows the power consumption of the active modes during the measurements and Tx/Rx transmission. The processor is active during these modes (processing, measurement, transmission and reception). All the remaining time to the next measurement, the processor is set to the Idle mode.

The average of power consumption in a given period P is presented in the following Formula (3)

(3) $$P_{aver}=\left(E_{proc}+E_{meas}+E_{Tx}+E_{Rx}+E_{Idle}\right)/P$$

$E_{proc},E_{meas},E_{Tx},E_{Rx},E_{Idle}$ are the energy dissipation of the different modes and which are represented in the following Equation (4).

(4) $$\begin{array}{c} \begin{array}{cc} E_{proc}=P_{proc}T_{proc} & ,\\ E_{meas}=\left(P_{proc}+P_{meas}\right)T_{meas} & ,\\ E_{Tx}=\left(P_{proc}+P_{Tx}\right)T_{Tx} & ,\\ E_{Rx}=\left(P_{proc}+P_{Rx}\right)T_{Rx} & ,\\ E_{Idle}=P_{Idle}T_{Idle} \end{array} \end{array}$$

According to Equations (3) and (4), the average of power consumption of the proposed system is equal to 13.48 mW. If we use a 2000 mAH battery, the system autonomy is up to one month. We notice that approximately 50% of the power consumption is caused by the processor chip during the active mode. Optimising the processor power consumption could enhance the system autonomy. Thus, more attention should be given to the micro-controller platform, especially the use of the sleep mode as well as the operating frequency.

## 8. Conclusions

In this paper, we proposed LoRaChainCare an IoT Architecture based on Blockchain technology for secure and authorized health data sharing, including patients vital signs and medical reports, by leveraging qualitative technologies. Blockchain, Edge/Fog computing and LoRaWAN were resorted to cope with the QoS requirements of HCS, comprising primarily low cost, security, scalability and reliable performance. Our proposed system allows health practitioner to monitor patients, ensuring health and medical safety are protected. The private-permissioned blockchain storage using decentralized file sharing system based on IPFS preserves security and solves Blockchain cost and scalability problem to share and store large data. Furthermore, our proposed HCS integrates Edge and Fog layers between IoT and Cloud layers. An Arduino Uno baord acts as an edge device and communicates with the Fog device through a LoRa communication protocol which preserves reliable performance. As a proof of concept, we implemented a full LoRaChainCare prototype. The implemented system integrates environmental and health sensors connected to Arduino Uno board that uses an ATmega328P microcontroller is embedded and equiped with LoRa shield. Also, a web-user application is developed to allow medical staff to explore Blockchain services or to upload high-storage report into IPFS. We integrate the IPFS storage along with Ethereum to store large data that would be cost heavy if stored on Blockchain.

The evaluation conducted in terms of cost analysis, runtime analysis and power consumption were performed using real IoT measurements. The cost analysis results have demonstrated a low gas cost for the overall Euthereum related services. The actual performance measurements show the efficiency of LoRaChainCare by ensuring a low runtime and power consumption required to send a sensor reading from the edge layer to the Blockchain. Powered by a 200 mAH battery, we estimate our system autonomy is up to one month.

Future research needs to be applied to optimize LoRaChainCare autonomy by optmizing microcontroller power consumption. Besides, for security and privacy concerns, integrating machine learning algorithms would be effective to add some HCS system prioritisation strategies. As with a vast, artificial intelligence and machine learning would be revolutionary tools to empower HCS with much more security and efficiency.

## Figures and Tables

**Table 1:** Comparative analysis of related works.

	Layers *	Communication Protocols	Blockchain	HCS Implementation	IPFS	Fog Devices
Ghosh et al. [30] (Mobi-IoST)	E-F-C	Not specified	X	X	X	Road Side Unit
Mukherjee et al. [31]	E-F-C	Not specified	X	✓	X	Smartphone
Gi et al. [29] (Edge-AI)	E-F-C	LoRa	X	X	X	LoRa AP
Ray et al. [15] (IoBHealth)	F-C	Zigbee-Bluetooth	✓	X	X	Smartphone
Sharma et al. [33]	F-C	Not specified	✓	X	X	laptop
Shukla et al. [34]	F-C	Not specified	X	X	X	RaspberryPi
Tuli et al. [35] (FogBus)	F-C	Zigbee-Bluetooth	✓	✓	X	Smartphone
Jamil et al. [13]	C	Wifi	✓	✓	X	X
Steichen et al. [37]	C	Not specified	✓	X	✓	X
Nguyen et al. [38]	F-C	Not specified	✓	✓	✓	SmartPhone
Md. Ashraf et al. [14]	E-F-C	ZigBee-Bluetooth	✓	X	X	Smartphone

* E = Edge layer, F = Fog layer, C = Cloud layer; X = Not adopted, ✓ = Adopted.

**Table 2:** Description of healthcare sensors connected to Arduino Uno.

	Sensor Description	Readings	Possible Disease
ECG AD8232	reads the cardiac electrical activity	PR interval	first degree of heart block early activation of ventricules
DHT11	sensing room temprature and humudity	PHR	bradicardia, tachycardia
SEN-11574	sensing heartbeat rate	H, T	defined by MS

**Table 3:** Smart Contract for the proposed LoRaChainCare.

Type	Components	Description
Asset	Sensors and readings	The health sensors and the related readings
Actors	MHSP	Hospital administrator
	Doctor	Medical staff
	Patient	User
	Nurse	Medical staff
Transactions	registerPatient	Transaction which is used to register a patient
	registerDoctor	Transaction which is used to register a doctor
	getPatientInfoForPatient	Transaction which allows patient to get the Patient’s stored information
	getPatientInfoForDoctor	Transaction which allows allowed doctor to get the patient’s stored information
	getPatientDataForPatient	Transaction which allows patient to get the Patient’s stored sensors data
	getDoctorInfo	Transaction which allows to get the doctor’s stored information
	getDoctorsList	Transaction which allows to get the list of doctors of a patient
	getPatientList	Transaction which allows to get the list of patients of a doctors
	grantAccessToDoctor	Transaction which is used to search for a patient
	addFileByPatient	Transaction which allows patient to add medical report
	addFileByDcAndMHSP	Transaction which allows MHSP and allowed doctors to add medical report to patient
	getFiles	Transaction which allows patient to get his medical report
	setEcg	Transaction to store the collected data from ECG sensor
	setTemperature	Transaction to store the collected data from Temperature sensor
	setHeartRate	Transaction to store the collected data from Heart Rate sensor
	getEcg	Transaction to get the stored data of ECG sensor
	getTemperature	Transaction to get the stored data of Temperature sensor
	getHeartRate	Transaction to get the stored data of Heart Rate sensor

**Table 4:** Gas Costs of the Smart Contract’s Functions.

Function Caller	Function Name	Gas Used	Cost in Ether	Cost in USD
MHSP	signupPatient	132,585	0.00265170	$10.70
MHSP	signupDoctor	131,283	0.00262566	$10.60
MHSP	grantAccessToDoctorByMHSP	107,183	0.00214366	$8.65
MHSP/Doctor	addFileByDcAndMHSP	276,361	0.00552722	$22.31
Patient	addFileByPt	136,018	0.00272036	$10.98
Patient	grantAccessToDoctor	76,746	0.00153492	$6.19
EcgSensor	setECG	74,740	0.00149480	$6.20
TemperatureSensor	setTemperature	74,733	0.00149466	$6.03
HeartRateSensor	setHeartRate	74,743	0.00149486	$6.03

**Table 5:** Transmission Time Details through all the Layers.

Process	Time	Process	Time
Starting Time	00:26:14	payload sent by the End Device	00:26:21.68
Sending Join Request	00:26:16.01	Payload in the Gateway	00:26:24.4575
Join request in the Gateway	00:26:18	Payload in the LoRa Server	00:26:24.5378
Join Accept sent by the LoRa server	00:26:20	Payload in the application Server	00:26:24.8549
join Accept received	00:26:21.65	Payload in The Blockchain	00:26:25

**Table 6:** Sx1276 Power characteristics.

Mode	Current Supply	Power Consumption
Idle	1.5 μA	4.95 μW
Transmission (Tx)	20 mA	66 mW
Receiver (Rx)	10.3 mA	34 mW

**Table 7:** Atmega328p Processor and sensors Power consumption.

Component	Current Supply	Power Consumption
Atmega328p	(Active) 9 mA	45 mW
	(Idle) 1.75 mA	8.75 mW
DHT11	0.3 mA	1 mW
AD8232	170 μA	561 μW
SEN-11574	3 mA	9.9 mW

**Table 8:** Power consumption and Time duration of active modes.

Mode	Power Consumption (mW)	Time Duration (s)
Active processor	45	5.68
Idle processor	8.75	54.32
Measurements	11.46	0.125
Transmission	66	0.58
Reception	34	1.12

## References

[1] Lv Z., Hu B., Lv H. (2020). Infrastructure Monitoring and Operation for Smart Cities Based on IoT System. IEEE Trans. Ind. Inform..

[2] Islam M., Rahaman A., Islam R. (2020). Development of Smart Healthcare Monitoring System in IoT Environment. SN Comput. Sci..

[3] Health Care. https://www.my.gov.sa/wps/portal/snp/aboutksa/HealthCareInKSA.

[4] Al-Fuqaha A., Guizani M., Mohammadi M., Aledhari M., Ayyash M. (2015). Internet of Things: A Survey on Enabling Technologies, Protocols, and Applications. IEEE Commun. Surv. Tutor..

[5] Connected Medical Device Market-Growth, Trends, Covid-19 Impact, and Forecasts (2021–2026). https://www.mordorintelligence.com/industry-reports/connected-medical-device-market.

[6] Hussien H.M., Yasin S.M., Udzir S.N.I., Zaidan A.A., Zaidan B.B. (2019). A Systematic Review for Enabling of Develop a Blockchain Technology in Healthcare Application: Taxonomy, Substantially Analysis, Motivations, Challenges, Recommendations and Future Direction. J. Med. Syst..

[7] Gordon W.J., Catalini C. (2018). Blockchain technology for healthcare: Facilitating the transition to patient-driven interoperability. Comput. Struct. Biotechnol. J..

[8] Abid A., Cheikhrouhou S., Kallel S., Jmaiel M. (2021). NovidChain: Blockchain-based privacy-preserving platform for COVID-19 test/vaccine certificates. Softw. Pract. Exper..

[9] Abid A., Cheikhrouhou S., Jmaiel M. (2020). Modelling and Executing Time-Aware Processes in Trustless Blockchain Environment. SRisks and Security of Internet and Systems, Proceedings of the 14th International Conference, CRiSIS 2019, Hammamet, Tunisia, 29–31 October 2019.

[10] Abid A., Cheikhrouhou S., Jmaiel M. Temporal constraints in smart contract-based process execution: A case study of organ transfer by healthcare delivery drone. Proceedings of the Volume 3067 of CEUR Workshop Proceedings, Tunisian-Algerian Joint Conference on Applied Computing (TACC 2021).

[11] Abid A., Cheikhrouhou S., Jmaiel M. How blockchain helps to combat trust crisis in COVID-19 pandemic? poster abstract. Proceedings of the 18th Conference on Embedded Networked Sensor Systems.

[12] Tsung-Ting Kuo H.E.K., Ohno-Machado L. (2017). Blockchain distributed ledger technologies for biomedical and health care applications. J. Am. Med. Inform. Assoc..

[13] Jamil F., Ahmad S., Iqbal N., Kim D.H. (2020). Towards a Remote Monitoring of Patient Vital Signs Based on IoT-Based Blockchain Integrity Management Platforms in Smart Hospitals. Sensors.

[14] Uddin M.A., Stranieri A., Gondal I., Balasubramanian V. (2020). Blockchain leveraged decentralized IoT eHealth framework. Internet Things.

[15] Ray P.P., Dash D., Salah K., Kumar N. (2021). Blockchain for IoT-Based Healthcare: Background, Consensus, Platforms, and Use Cases. IEEE Syst. J..

[16] Abdellatif A.A., Mohamed A., Chiasserini C.F., Tlili M., Erbad A. (2019). Edge Computing for Smart Health: Context-Aware Approaches, Opportunities, and Challenges. IEEE Netw..

[17] Dong P., Ning Z., Obaidat M.S., Jiang X., Guo Y., Hu X., Hu B., Sadoun B. (2020). Edge Computing Based Healthcare Systems: Enabling Decentralized Health Monitoring in Internet of Medical Things. IEEE Netw..

[18] Pace P., Aloi G., Gravina R., Caliciuri G., Fortino G., Liotta A. (2019). An Edge-Based Architecture to Support Efficient Applications for Healthcare Industry 4.0. IEEE Trans. Ind. Inform..

[19] Kumari A., Tanwar S., Tyagi S., Kumar N. (2018). Fog computing for Healthcare 4.0 environment: Opportunities and challenges. Comput. Electr. Eng..

[20] Flavio B., Rodolfo M., Jiang Z., Sateesh A. (2015). Fog Computing and the Internet of Things: Extend the Cloud to Where the Things Are.

[21] Filho I.d.M.B., Aquino G., Malaquias R.S., Girão G., Melo S.R.M. (2021). An IoT-Based Healthcare Platform for Patients in ICU Beds During the COVID-19 Outbreak. IEEE Access.

[22] Shi Y., Ding G., Wang H., Roman H.E., Lu S. The fog computing service for healthcare. Proceedings of the 2015 2nd International Symposium on Future Information and Communication Technologies for Ubiquitous HealthCare (Ubi-HealthTech).

[23] Semtech (2019). LoRa and LoRaWAN: A Technical Overview. https://lora-developers.semtech.com/uploads/documents/files/LoRa_and_LoRaWAN-A_Tech_Overview-Downloadable.pdf.

[24] Puthal D., Malik N., Mohanty S.P., Kougianos E., Das G. (2018). Everything You Wanted to Know About the Blockchain: Its Promise, Components, Processes, and Problems. IEEE Consum. Electron. Mag..

[25] Aazam M., Huh E.N. Dynamic resource provisioning through Fog micro datacenter. Proceedings of the 2015 IEEE International Conference on Pervasive Computing and Communication Workshops (PerCom Workshops).

[26] Wang D., Wang P. (2018). Two Birds with One Stone: Two-Factor Authentication with Security Beyond Conventional Bound. IEEE Trans. Dependable Secur. Comput..

[27] Rahmani A.M., Gia T.N., Negash B., Anzanpour A., Azimi I., Jiang M., Liljeberg P. (2018). Exploiting smart e-Health gateways at the edge of healthcare Internet-of-Things: A fog computing approach. Future Gener. Comput. Syst..

[28] Verma P., Sood S.K. (2018). Cloud-centric IoT based disease diagnosis healthcare framework. J. Parallel Distrib. Comput..

[29] Queralta J.P., Gia T.N., Tenhunen H., Westerlund T. Edge-AI in LoRa-based Health Monitoring: Fall Detection System with Fog Computing and LSTM Recurrent Neural Networks. Proceedings of the 2019 42nd International Conference on Telecommunications and Signal Processing (TSP).

[30] Ghosh S., Mukherjee A., Ghosh S.K., Buyya R. (2020). Mobi-IoST: Mobility-Aware Cloud-Fog-Edge-IoT Collaborative Framework for Time-Critical Applications. IEEE Trans. Netw. Sci. Eng..

[31] Mukherjee A., Ghosh S., Behere A., Ghosh S.K., Buyya R. (2021). Internet of Health Things (IoHT) for personalized health care using integrated edge-fog-cloud network. J. Ambient. Intell. Humaniz. Comput..

[32] Sabella D., Sukhomlinov V., Trang L., Kekki S., Paglierani P., Rossbach R., Li X., Fang Y., Druta D., Giust F. (2019). Developing Software for Multi-Access Edge Computing. ETSI White Pap..

[33] Sharma P.K., Chen M.Y., Park J.H. (2018). A Software Defined Fog Node Based Distributed Blockchain Cloud Architecture for IoT. IEEE Access.

[34] Shukla S., Thakur S., Hussain S., Breslin J.G., Jameel S.M. (2021). Identification and Authentication in Healthcare Internet-of-Things Using Integrated Fog Computing Based Blockchain Model. Internet Things.

[35] Tuli S., Mahmud R., Tuli S., Buyya R. (2019). FogBus: A Blockchain-based Lightweight Framework for Edge and Fog Computing. J. Syst. Softw..

[36] Ekblaw A., Azaria A., Halamka J.D., Lippman A. (2010). A Case Study for Blockchain in Healthcare: “MedRec” Prototype for Electronic Health Records and Medical Research Data.

[37] Steichen M., Fiz B., Norvill R., Shbair W., State R. Blockchain-Based, Decentralized Access Control for IPFS. Proceedings of the 2018 IEEE International Conference on Internet of Things (iThings) and IEEE Green Computing and Communications (GreenCom) and IEEE Cyber, Physical and Social Computing (CPSCom) and IEEE Smart Data (SmartData).

[38] Nguyen D.C., Pathirana P.N., Ding M., Seneviratne A. (2019). Blockchain for Secure EHRs Sharing of Mobile Cloud Based E-Health Systems. IEEE Access.

[39] Chen Y., Li H., Li K., Zhang J. An improved P2P file system scheme based on IPFS and Blockchain. Proceedings of the 2017 IEEE International Conference on Big Data (Big Data).

[40] Fox S., Naughton J.H.W. (1971). Physical activity and the prevention of coronary heart disease. Ann. Clin. Res..

[41] Bhaskar Kashyap (2021). Introduction to Smart Contracts. https://ethereum.org/en/developers/docs/smart-contracts/.

[42] Remix-Ethereum IDE. https://remix.ethereum.org/.

[43] React—A JavaScript Library for Building User Interfaces. https://reactjs.org/.

[44] MetaMask-A Crypto Wallet & Gateway to Blockchain Apps. https://metamask.io/.

[45] Ganache. https://trufflesuite.com/ganache/.

[46] Semtech (2015). SX1276 Development Kit User Guide. https://semtech.my.salesforce.com/sfc/p/#E0000000JelG/a/2R0000001Rbr/6EfVZUorrpoKFfvaF_Fkpgp5kzjiNyiAbqcpqh9qSjE.

[47] Atmel (2015). Atmega328p Datasheet. https://ww1.microchip.com/downloads/en/DeviceDoc/Atmel-7810-Automotive-Microcontrollers-ATmega328P_Datasheet.pdf.

[48] ADSONG DHT11 Datasheet. https://components101.com/sites/default/files/component_datasheet/DHT11-Temperature-Sensor.pdf.

[49] Analog Devices Single-Lead, Heart Rate Monitor Front End. https://cdn.sparkfun.com/datasheets/Sensors/Biometric/AD8232.pdf.

[50] SEN-11574 Heart Rate Sensor Datasheet. https://media.digikey.com/pdf/Data%20Sheets/Pulse%20Sensor%20PDFs/Pulse_Sensor.pdf.

[51] Bouguera T., Diouris J.F., Chaillout J.J., Jaouadi R., Andrieux G. (2018). Energy consumption model for sensor nodes based on LoRa and LoRaWAN. Sensors.

